# Effectiveness of OH card-based group mental health education in improving mood and behavior in breast cancer patients

**DOI:** 10.3389/fpubh.2025.1533073

**Published:** 2025-03-14

**Authors:** Zhaojun Wang, Xiaoqing Hu, Jiangang Xu, Jieyu Zhou, Xiaojing Ou, Meihua Chen

**Affiliations:** ^1^Department of Thyroid and Breast Surgery, The Wenzhou Central Hospital, The DingLi Clinical College of Wenzhou Medical University, Wenzhou, Zhejiang, China; ^2^Department of Rehabilitation, The Wenzhou Central Hospital, The DingLi Clinical College of Wenzhou Medical University, Wenzhou, Zhejiang, China

**Keywords:** breast cancer, HADS, SCSQ, anxiety, OH card

## Abstract

**Purpose:**

Psychological disorders and different coping styles often occur after breast cancer (BC) diagnosis. The purpose of this study was to explore the effect of OH card on psychological status and coping styles of individuals with breast cancer.

**Methods:**

This was a non-randomized trial in which 54 outpatients or inpatients with BC who were willing to be assessed using psychological scales, allocated to either the OH card intervention group (OHG) or the usual care group (CG). The OHG received 1 session of OH card therapy over 2 h. Participants completed assessments of anxiety and depressive symptoms and coping styles using the Hospital Anxiety and Depression Scale (HADS) and Simplified Coping Style Questionnaire (SCSQ) scales at baseline, month 1, 3 and 6 post-intervention. Data were analyzed using descriptive statistics, chi-squared test and repeated measures ANOVA.

**Results:**

The HADS score in the intervention group was lower than that of the control group by 2.296 (*p*<0.05) at 1 month post-intervention. The SCSQ-positive coping aspect of usual care group scores showed a downward trend, while the OH card intervention group scores showed an upward trend, with a significant difference between the two groups (*p* = 0.040), and the difference between the two groups was significant at 1, 3 and 6 months after the intervention (all *p* < 0.05).

**Conclusion:**

The results of our study suggest that OH card intervention may improve symptomatology of anxiety and depression among people with BC at month1, and promote positive behavior within 6 months. The OH card intervention has a potential role in the psychological rehabilitation of individuals with breast cancer and warrants further research.

## Introduction

Breast cancer stands as the predominant global cancer and the primary cause of cancer-related mortality among women (1). Research indicates that a significant proportion, ranging from 20 to 50% of women, undergo psychological distress throughout the diagnosis and treatment phases of breast cancer. The diagnosis alone serves as a detrimental trigger, compounded by postoperative bodily alterations, treatment repercussions, and occupational and financial stressors, intensifying both physical and psychological distress (2). Anxiety, distress, depression, and post-traumatic stress disorder rank among the prevailing psychological conditions observed in individuals grappling with breast cancer (3). The prevalence of anxiety and depression is significantly higher among breast cancer survivors compared with the general population, with variations observed across different regions and times after diagnosis (4–6). Notably, 19.4% of patients endure severe psychological distress within the first year post-diagnosis, while the overall prevalence of depression reaches 30.2%, correlating strongly with advanced disease stages (7–9). Even 6 months post-surgery and completion of adjuvant therapy, patients may still grapple with abnormal psychological states such as low self-efficacy, distress, anxiety, and depression (10). Research underscores the pivotal influence of depression and anxiety as autonomous prognosticators for cancer relapse and longevity (11), highlighting the urgency for timely interventions.

Current interventions, such as educational programs, positive thinking therapy, and exercise interventions, aim to mitigate distress and enhance quality of life (12–14). However, barriers such as treatment-related fatigue, cognitive overload, and reliance on professional guidance limit their sustainability and accessibility. Coping strategies, characterized as the mechanism through which individuals harmonize their internal equilibrium with the external milieu by modifying their cognition and conduct (15), can either ameliorate or exacerbate depression symptoms based on the positivity or negativity of coping strategies (16). Coping strategies play a crucial role in mitigating psychological distress encompassing depression, anxiety, and stress among cancer patients (17), enhancing these strategies through targeted interventions proves critical for improving clinical outcomes in breast cancer populations.

In China, where cultural stigma often deters patients from seeking psychiatric care. Consequently, the oncology team typically plays a more important role in furnishing patients with psychological and spiritual sustenance. Group therapy has demonstrated efficacy across various psychiatric disorders and has exhibited advantageous outcomes for individuals grappling with physical ailments. Diverse therapeutic modalities have been extended to people with breast cancer within a group framework, proving efficacious. These include psychoeducation, cognitive behavioral therapy, supportive-expressive group therapy, and mindfulness-based therapy (18–23). Furthermore, multiple meta-analyses have shown the significant efficacy of group therapy in diminishing psychological distress and enhancing patients’ coping mechanisms and adaptive proficiencies (12, 24). Patients’ feelings of stigma and isolation stemming from their illness can be reduced by engagement in group activities. Moreover, providing reliable, evidence-based information regarding the disease can assuage anxiety and foster a perception of proficiency in managing a severe condition like breast cancer. Through the exchange of coping experiences related to illness and treatment hurdles in a group environment, patients can emerge as exemplars for fellow individuals, thereby instilling hope (12).

The OH Card, known as a “subconscious projection card, “was developed in the early 1980s by Moritz Egetmeyer, a German humanistic psychologist, and Ely Raman, a Mexican-born artist. The deck comprises 88 image cards and 88 word cards. Users explore subconscious thoughts and emotions by randomly drawing cards and interpreting their meanings. OH cards help individuals to understand their inner world and emotional needs better, promote their self-growth and development, improve interpersonal relationships, and improve their mental health by using the principles of projection technology, unconscious expression, symbolic meaning, free association, emotional expression and release, deep insight, and interpersonal interaction analysis (25). As a projective test, OH Cards have demonstrated clinical utility in individual counseling sessions and group therapy for nursing personnel. Through therapeutic interactions, participants engage in self-reconstitution, release stress, alleviate anxiety symptoms, and formulate actionable implementation plans under guided facilitation (26–28). Yuna Jiang reported OH Cards can increase the posttraumatic growth scores of children with fractures, improve their coping styles, reduce stress disorders, decrease depression and improve their psychological state and promote their recovery (29). Despite their widespread use, the application of OH cards in psychotherapy for oncology patients remains unexplored.

In light of this gap, the current research team posited that the psychological insights derived from OH cards interventions may offer therapeutic benefits for patients experiencing tumor-related psychological distress. This study aims to investigate the impact of OH card therapy on anxiety, depression, and behavioral patterns among people with breast cancer. This pioneering endeavor represents a significant step toward offering a novel avenue for psychological rehabilitation in breast cancer care, laying the groundwork for clinical interventions in this domain.

## Methods

### Study design

**Figure 1:**
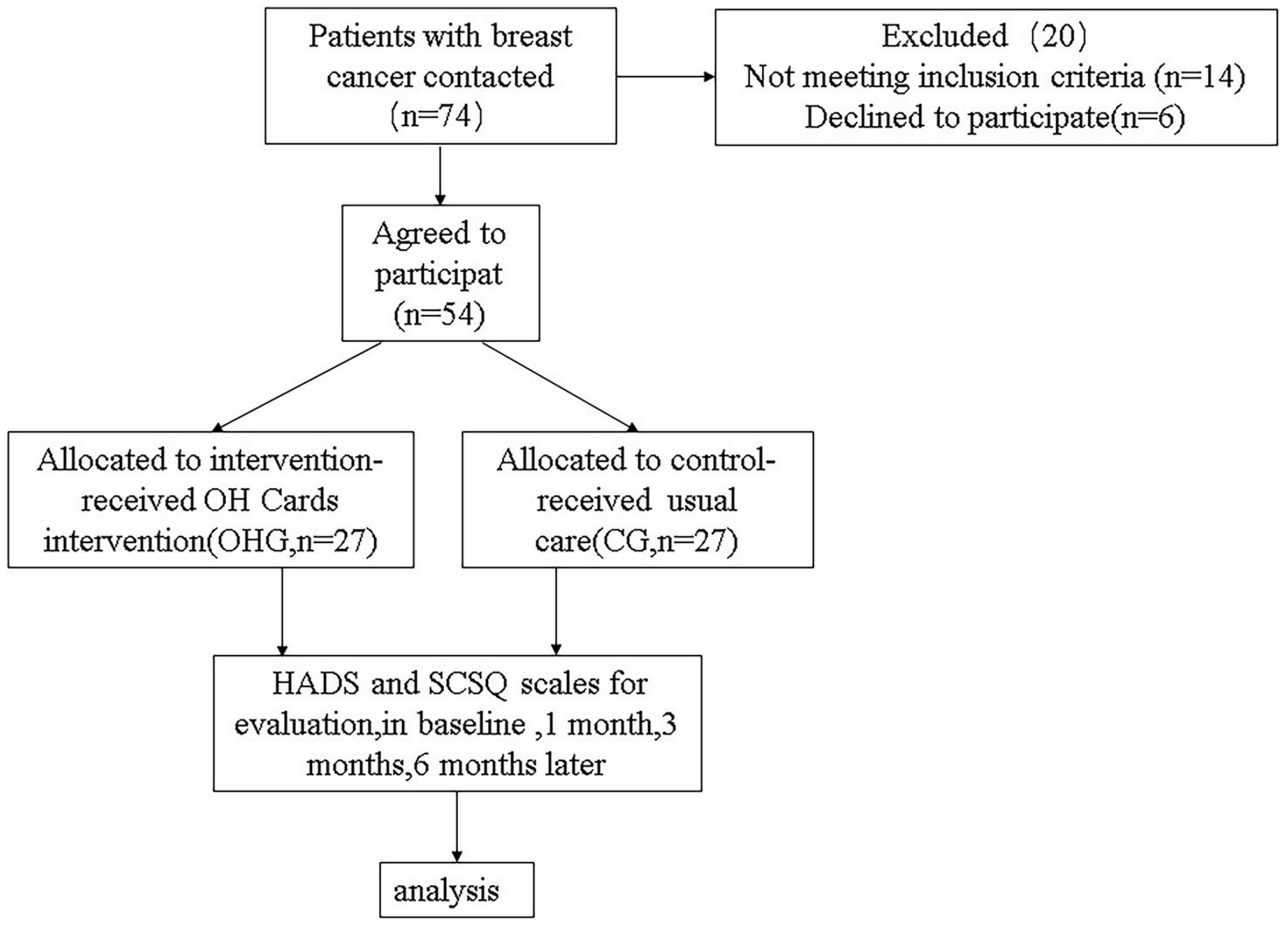
Flowchart of the sample of patients with breast cancer. CG, usual care group; OHG, OH card intervention group.

This was a prospective longitudinal study (Figure 1) and the team delivering the program included a clinical psychologist and experienced nurses. Given significant individual variations in patients’ receptiveness to psychological interventions, coupled with the fact that some patients actively opted out of participation due to cultural beliefs (e.g., stigma against psychotherapy), we implemented a non-randomized controlled design to respect patient autonomy and enhance compliance. The study was approved by the Ethics Committee of Wenzhou Central Hospital (approval number 202305222305000391700) and was conducted in accordance with the ethical standards of the Declaration of Helsinki.

A power analysis using G*Power 3.1.9.7 software indicated 42 participants were required (21 per group) to detect an effect of moderate magnitude (*F* = 0.25; *α*-error = 0.05, power = 0.8, groups = 2, number of measurements = 4, correlation among general linear measures: *r* = 0.50). To counterbalance possible dropouts, the total sample size was set at 74 individuals (=37/condition), and 54 patients completed the study.

### Participants

Cases who were treated as outpatients or inpatients in our hospital from 1 June to 15 June 2023, and who were willing to be assessed using the psychological scale, were selected. Inclusion criteria: ① aged≥18 years; ② pathologically confirmed as breast cancer; ③ literacy level above primary school; ④ HADS score ≥ 8; ⑤ patients themselves are aware of the study and sign the informed consent form. Exclusion criteria: ① patients with other mental illnesses and serious physical illnesses; ② pregnant or lactating women; ③ people with speech communication and comprehension disorders; ④ people with bipolar disorder or with alcohol, drug or other substance abuse; ⑤ patients prior exposure to similar intervention protocol.

### Procedures

In this study, a cohort of 27 patients voluntarily engaged in OH card therapy, with participants self-selecting whether to be part of the experimental group. Patients enrolled in the study received usual care in addition to the intervention. The intervention group underwent a single session of OH card therapy. Assessment points were scheduled at intervals: 1 week before the treatment initiation, and then at month 1, month 3, and month 6 post-treatment. Adverse events (e.g., mood deterioration) were monitored throughout the study, with no participants withdrawing due to intervention-related discomfort.

Usual care in this context entails the provision of comprehensive support, including breast cancer education, psychological reassurance, safety information, and dietary guidance throughout the treatment process.

### Intervention

The intervention group, consisting of 9 patients per session, engaged in a 2-h OH card therapy session structured into four distinct phases: the start phase, the introduction phase, the work phase, and the end phase. The psychological counselor from Wenzhou Seventh People’s Hospital who are familiar with the principles and skills of OH cards and knows how to use them in consultation provided psychological counseling. The venue was arranged in a warm and bright area making people feel safe and comfortable located within the hospital outpatient building that was not yet open to the public.

Start Phase: This phase commenced by outlining the game’s rules, emphasizing the importance of respecting others and safeguarding privacy. The primary goal was to cultivate a secure and empathetic environment conducive to sharing, aimed at relieving group members’ anxiety. The leader guided participants to choose an OH card representing their roles, introduce themselves and explain their choice in the group. Subsequently, they picked an OH card and expressed their current emotions, intentions, and expectations for participating in the activity and find similarities between themselves and this card. Use these two cards to introduce themselves in the group and promote mutual understanding among members.

Introduction Phase: Referred to as the “words-only” phase, each individual drew an OH card and described it using an adjective. The cards were then concealed, thoroughly shuffled by the leader, and subsequently revealed to identify the corresponding participant.

Work Phase: During this stage, three cards were drawn at random, symbolizing the person’s past, present, and future. Participants shared their innermost feelings, fostering self-exploration, mutual support, and enlightenment among group members.

Closing Phase: The session concluded with a review of character and OH cards to reflect on initial expectations, share unexpected insights, and discuss reactions to the session’s conclusion.

### Measures

Measurements were made at baseline, month 1, month 3, month 6 following the intervention using Wen Juan Xing (a professional online platform for questionnaire surveys, exams, evaluations, and voting). (1) The HADS assessed their psychological state. Score description: The scores of the two subscales of anxiety (HADS-A) and depression (HADS-D) were divided as follows: 0–7 as negative; 8–10 as mild; 11–14 as moderate; and 15–21 as severe (30). (2) SCSQ: In this study, we used the revised and improved SCSQ by Jie (31) to assess patients’ cognitive and behavioral styles in the face of stress, including two dimensions: positive coping and negative coping. The questionnaire consists of 20 items scored on a 4-point scale from “never use” to “often use” on a 0–3 scale. Items 1–12 assess positive coping and items 13–20 focus on assessing negative coping characteristics. The higher the score, the more the individual tends to use coping styles.

In this study, the Cronbach alpha for this total scale, HADS, SCSQ, respectively, was 0.75, 0.78, and 0.79.

### Data analysis

The research team analyzed the data using SPSS 22 statistical software. The research team (1) expressed count data as numbers (N) and percentages (%) and compared between groups using the chi-squared χ^2^ test. (2) Score of HADS, SCSQ measurements were expressed as mean ± standard deviation (SD), then repeated measures analysis of covariance (ANCOVA) implemented under a general linear model. If there was significant difference at the treatment time interaction, multiple comparisons are performed between the two groups, at different time points. The data were evaluated using Bonferroni’s adjustment to control for multiple testing. Differences were deemed significant when *p* < 0.008.

## Results

### Baseline characteristics

The study sample comprised 74 patients with BC. Of these, 54 patients agreed to participate; all completed the instruments at baseline, month 1, month 3, month 6 following the intervention. There was no significant difference between the two groups in baseline demographic data and clinical characteristics at the baseline (Table 1).

**Table 1:** Participant characteristics (*n* = 54).

Characteristics	Control group (CG); *n* = 27; n (%)	Intervention group (OHG); *n* = 27; n (%)	χ^2^/t	OR(95%CI)	*p* value
Surgical unit			2.308	2.588 (0.744–9.000)	0.129
Our hospital	22	17			
Others	5	10			
Type of breast surgery			0.429	0.649 (0.177–2.377)	0.513
Mastectomy	20	22			
Breast conserving therapy	7	5			
Type of axillary treatment			0.731	0.612 (0.198–1.892)	0.393
Axillary lymph node dissection	16	19			
Sentinel node procedure	11	8			
Age, y			0.595	1.259(−2.986–5.504)	0.554
Years	50.19 ± 7.62	48.93 ± 7.93			
Laterality			1.187	1.818 (0.618–5.352)	0.414
Right	15	11			
Left	12	16			
Education Level			0.297	0.743 (0.255–2.167)	0.586
Primary	12	14			
High school or above	15	13			
Employment Status			1.964	0.357 (0.082–1.564)	0.161
Employed	3	7			
Unemployed	24	20			
Stage			0.554	/	0.963
I	9	7			
II	8	8			
III	6	7			
IV	4	5			
Time since diagnosis (in months)			0.667	0.064 (0.219–1.872)	0.414
≤6	12	15			
>6	15	12			
ER			0.300	1.351 (0.460–3.964)	0.584
Negative	16	14			
Positive	11	13			
PR			0.076	0.859 (0.292–2.530)	0.783
Negative	15	16			
Positive	12	11			
Her-2			0.731	0.612 (0.198–1.892)	0.393
Negative	16	19			
Positive	11	8			
Ki-67			0.000	1.000 (0.323–3.101)	1.000
≤20%	9	9			
>20%	18	18			
Subtyping			1.028	/	0.869
Luminal A	2	3			
Luminal B	12	14			
Her-2 positive	5	3			
Triple negative	8	7			
Type of treatment			4.542	/	0.515
Perioperative	5	2			
Under Chemotherapy	11	9			
Under endocrine	6	8			
Under targeted therapy	2	2			
Under Radiotherapy	0	3			
No treatment	3	3			

OR, odds ratio.

### Represents change in anxiety and depression

#### HADS-A

Baseline HADS-A scores in the CG and OHG were 7.370 ± 2.937 and 7.440 ± 3.735 (*t* = −0.081, *p* = 0.936; Table 2).

**Table 2:** Mean score, standard deviation, *p* value, Cohen‘s d of HADS, at the baseline, month 1, month 3, month 6 after the intervention.

Outcomes	Time	Groups		F	*p*	Cohen‘s d^*^	95% CI
		CG (mean ± SD)	OHG (mean ± SD)				
HADS-A	Baseline	7.370 ± 2.937	7.440 ± 3.735	0.007	0.936		
	Month 1	8.667 ± 2.801^a^	7.148 ± 2.85	3.897	0.054	0.54	[−0.005, 1.08]
	Month 3	8.296 ± 3.036	7.37 ± 3.702	1.010	0.320	0.27	[−0.26, 0.81]
	Month 6	8.222 ± 2.833	7.296 ± 3.729	1.055	0.309	0.28	[−0.26, 0.81]
	(F,*p*)^▲^	(4.103,0.011)	(0.252,0.860)				
HADS-D	Baseline	7.481 ± 2.310	7.111 ± 3.566				
	Month 1	8.444 ± 3.355	7.000 ± 3.101				
	Month 3	7.111 ± 2.225	6.593 ± 2.912				
	Month 6	7.296 ± 2.509	6.663 ± 2.976				
HADS	Baseline	14.852 ± 3.949	14.556 ± 6.272	0.043	0.863		
	Month 1	16.444 ± 3.401^a^	14.148 ± 4.737	4.187	0.046	0.57	[0.02, 1.11]
	Month 3	15.407 ± 3.775	13.555 ± 4.371	2.776	0.102	0.45	[−0.09, 0.99]
	Month 6	15.111 ± 3.445^b^	13.925 ± 5.348	0.937	0.338	0.26	[−0.27, 0.80]
	(F,*p*)	(5.075,0.004)	(1.050,0.379)				

CG, Control Group; OHG, OH card intervention group; HADS, Hospital Anxiety and Depression Scale; SD, standard deviation; HADS-A, HADS Anxiety; HADS-D HADS depression. Bonferroni-corrected pairwise comparisons between groups. *Values are absolute Cohen effect sizes of between-group differences. ▲Repeated measures ANOVA F-value and *p* of within-group differences.

a Significant difference from Baseline, *p* < 0.05.

b Significant difference from baseline Month 1 (*p* < 0.05).

Compared to baseline, mean anxiety scores in the CG increased by 1.297 points at month 1, a statistically significant change (*p* = 0.005), by 0.926 points at month 3 and by 0.852 points at month 6, a statistically insignificant change (*p* > 0.05).

The mean HADS-A score of the OHG was slightly reduced by 0.296 from baseline in the first month, and there was no significant reduction thereafter, and the change between time periods was not statistically significant (*p* > 0.05).

**Figure 2:**
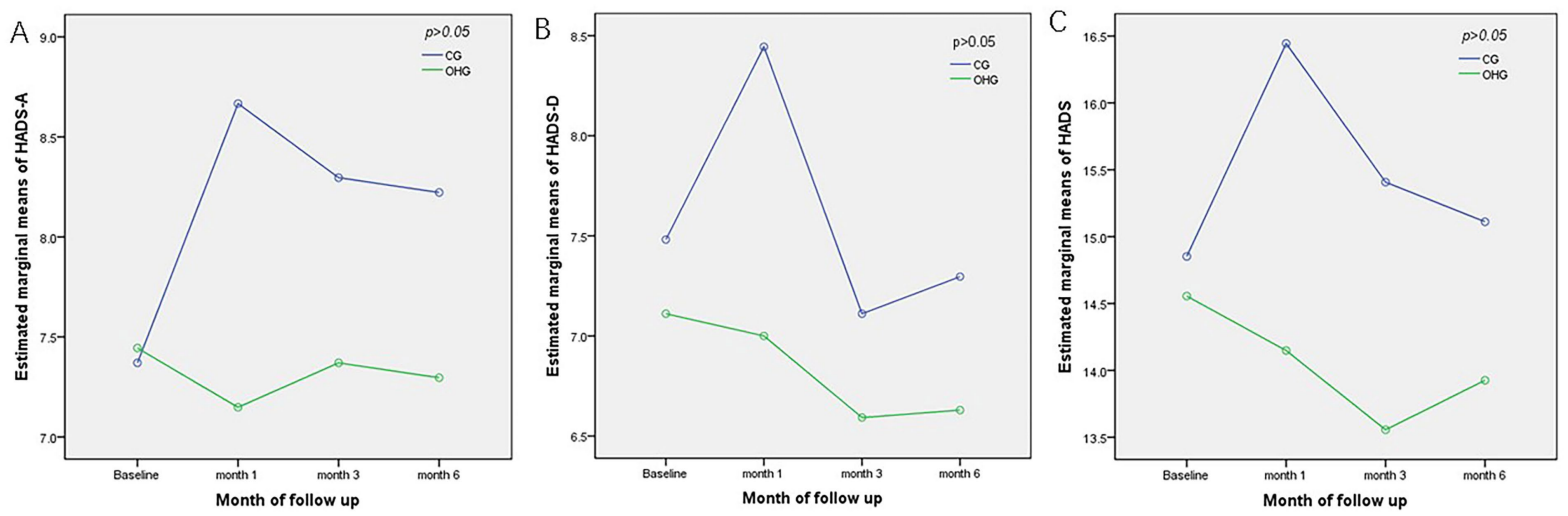
Comparison of estimated marginal means for HADS at all points of assessment between OHG and CG. **(A)** Estimated marginal means of HADS-A; **(B)** Estimated marginal means of HADS-D; **(C)** Estimated marginal means of HADS.CG, Control Group; OHG, OH card intervention group; HADS-A, HADS Anxiety; HADS-D HADS depression.

Multivariate analysis showed no statistically significant effect of the OH card intervention on patients’ anxiety status (*F* = 1.029, *p* = 0.315), overall time effects (*F* = 1.287, *p* = 0.289), and the interaction term between groups and time was statistically significant (*F* = 3.067, *p* = 0.036), confirming that different psychoeducational methods have different effects on patients’ anxiety status over time (Table 2; Figure 2).

#### HADS-D

Mean HADS-D scores at baseline were 7.481 ± 2.310 and 7.111 ± 3.566 for the CG and OHG, respectively (*t* = −0.453, *p* = 0.653; Table 2); mean HADS-D in the CG increased slightly at month 1 compared to baseline (*d* = 0. 963, *p* = 0.145) and decreased at month 3 and 6 (*d* = −0.370, *p* = 1.000; *d* = −0.185, *p* = 1.000), with a significant decrease in score at month 3 compared to month 1 (*d* = −1.333, *p* = 0.019).

Mean HADS-D scores in the OHG were slightly lower than baseline at month 1 (−0.111) and decreased to varying degrees thereafter, but the change between groups was not statistically significant (*p* > 0.05).

The between-group analysis revealed that the OH card intervention did not yield a statistically significant impact on the HADS-D scores (*F* = 1.173, *p* = 0.284). However, there was a noticeable trend of decreasing HADS-D scores over time, with the scores reaching their lowest point at the 3-month mark (*p* = 0.036), overall time effects (*F* = 1.099, *p* = 0.358), no pairwise comparisons were conducted.

Overall, mean HADS scores at baseline were 14.852 ± 3.949 and 14.556 ± 6.272 for the CG and OHG (*F* = 0.043, *p* = 0.836; Table 2). At month 1, the OHG score decreased to 2.296 compared to the CG (*p* < 0.05), and thereafter the intervention group’s total score was lower, but the difference between the groups was not statistically significant (*F* = 1.503, *p* = 0.226), overall time effects (*F* = 2.999, *p* = 0.085), interaction effects (*F* = 2.999, *p* = 0.032), then pairwise comparisons were conducted based on simple effects analysis. (Table 2; Figure 2).

Using G*Power 3.1.9.7 for *post-hoc* testing, power was 58% to detect a moderate Cohen‘s d effect size of 0.28 with this sample size, lower than the conventional standard (0.80).

### Represents change in coping style

**Figure 3:**
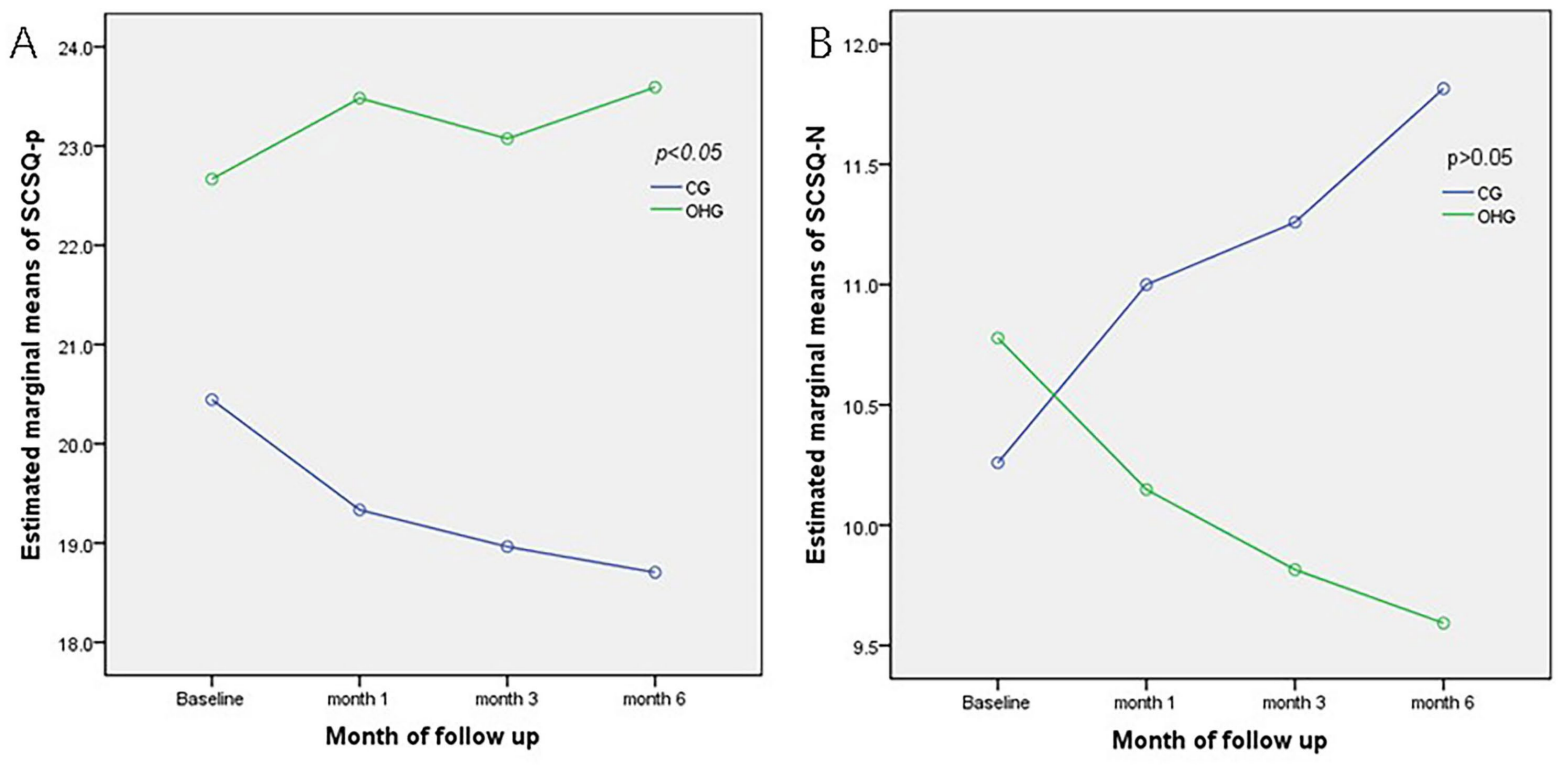
Comparison of estimated marginal means for SCSQ at all points of assessment between OHG and CG. **(A)** Estimated marginal means of SCSQ-p; **(B)** Estimated marginal means of SCSQ-n. CG, Control Group, OHG, OH card intervention group, SCSQ-N, SCSQ-Negative coping, SCSQ-P, SCSQ-Positive coping.

**SCSQ-Positive Coping**: At baseline, the mean scores were 20.444 ± 7.428 for the CG and 22.667 ± 7.711 for the OHG (*F* = 1.163, *p* = 0.286; refer to Table 3). Over time, the CG scores displayed a decreasing trend, especially a significant decrease at month 6 compared to baseline (*d* = 1.740, *p* = 0.042), while the OHG scores showed a tendency to increase. The disparity between the two groups was significant (*F* = 4.444, *p* = 0.040), with this distinction being notable at all time points (all *p* < 0.05; Figure 3). Overall time effects (*F* = 0.757, *p* = 0.523), interaction effects (*F* = 0.3.886, *p* = 0.015), then pairwise comparisons were conducted based on simple effects analysis. (Table 3).

**Table 3:** Mean score, standard deviation, *p* value, Cohen‘s d of SCSQ, at the baseline, month 1, month 3, month 6 after the intervention.

Outcomes	Time	Groups		F	*p*	Cohen‘s d	95% CI
		CG (mean ± SD)	OHG (mean ± SD)				
SCSQ-P	Baseline	20.444 ± 7.428	22.667 ± 7.711	1.163	0.286		
	Month 1	19.333 ± 6.403	23.482 ± 7.495	4.781	0.033	−0.60	[−1.15, 0.04]
	Month 3	18.963, SD6.105	23.074 ± 6.586	5.658	0.021	−0.65	[−1.19, 0.09]
	Month 6	18.704 ± 6.170^a^	23.593 ± 6.919	7.510	0.008	−0.75	[−1.30, 0.20]
	(F,*p*)	(2.554,0.066)	(2.066,0.117)				
SCSQ-N	Baseline	10.259 ± 4.679	10.778 ± 4.173	0.185	0.669		
	Month 1	11.000 ± 4.844	10.148 ± 3.820	0.515	0.476	0.20	[−0.35,0.75]
	Month 3	11.259 ± 4.888	9.815 ± 4.151	1.0.370	0.247	0.33	[−0.22, 0.88]
	Month 6	11.815 ± 4.946 ^a^	9.593 ± 4.060	3.256	0.077	0.52	[−0.04, 1.08]
	(F,*p*)	(3.266,0.029)	(1.704,0.178)				

CG, Control Group, OHG, OH card intervention group, SCSQ-N, SCSQ-Negative coping, SCSQ-P, SCSQ-Positive coping. Bonferroni-corrected pairwise comparisons between groups. *Values are absolute Cohen effect sizes of between-group differences. ▲Repeated measures ANOVA F-value and *p* of within-group differences.

a Significant difference from Baseline, *p* < 0.05.

**SCSQ-negative coping**: At baseline, the mean scores were 10.259 ± 4.679 for the CG and 10.778 ± 4.173 for the OHG (*F* = −0.430, *p* = 0.669; refer to Table 3). The CG exhibited an increasing trend in scores, while the OHG displayed a decreasing trend over time. However, the difference between the two groups was not statistically significant (*F* = 0.731, *p* = 0.396). Notably, the usual care group showed a significant increase at month 6 compared to baseline (*d* = 1.556, *p* = 0.026; Figure 3). Overall time effects (*F* = 0.287, *p* = 0.835), interaction effects (*F* = 4.682, *p* = 0.006), then pairwise comparisons were conducted based on simple effects analysis (Table 3).

Using G*Power 3.1.9.7 for *post-hoc* testing, based this sample size from **SCSQ-Positive Coping**, power was 93% to detect a moderate Cohen‘s d effect size of 0.47, higher than the conventional standard (0.80). However, power was 69% to detect a moderate Cohen‘s d effect size of 0.32 from SCSQ- negative Coping, lower than the conventional standard (0.80).

## Discussion

To the best of our knowledge, this clinical trial represents the inaugural attempt to intervene in mood regulation and coping strategies among people with breast cancer through the innovative use of OH cards.

In this study, changes in anxiety, depression, and coping styles were explored in 54 patients following an OH card intervention. Compared to usual care, the OH card intervention treatment notably enhanced emotional distress among people with breast cancer at month 1 post-intervention. However, its effectiveness was not sustained in the assessments conducted at month 3 and 6 thereafter. Furthermore, the results of HADS anxiety subscale and depression subscale did not demonstrate a reduction in distress levels at any time point or between the groups. This could be attributed to the natural decrease in anxiety and depression symptoms over time (32). Meanwhile, 50% of the patients enrolled in this study were non-surgical/chemotherapy patients, which also reflects the milder side effects associated with endocrine, targeted or radiotherapy. This demographic composition could have influenced the overall outcomes and responses observed in the study, particularly in relation to the effectiveness of the OH card intervention in managing anxiety-depression symptoms. Additionally, the intervention group received usual care during treatment, which encompassed breast cancer education, psychological support, safety guidance, and dietary advice-all of which have proven effective in alleviating anxiety and depression (14). Overall, only a decreasing trend in anxiety and depression scores was observed in the OH card intervention group for the reasons mentioned above. However, in the context of increased anxiety scores compared to the control group in the first and sixth months after enrolment, it may be useful to organize the activity single-session in this experiment. Obviously, there is a lack of intervention intensity or follow-up support. The prolonged treatment duration associated with comprehensive breast cancer therapy (typically 6–12 months or more) and potential dynamic psychological adaptation processes. Future research should explore multi-session intervention programs to sustain long-term effects.

The primary outcome of this study, as indicated by the SCSQ, revealed a significant enhancement in positive coping style scores at months 1, 3, and 6 post-intervention in the intervention group versus the control group. Significant between-group differences were observed across post-intervention assessments (month 1: *d* = −0.60[−1.15, 0.04]; month 3: *d* = −0.65[−1.19, 0.09]; month 6: *d* = −0.75[−1.30, 0.20]), consistent with moderate effect sizes per Cohen’s criteria. This suggests that the utilization of OH cards can effectively enhance the positive coping mechanisms among individuals with breast cancer. Wang et al. demonstrated that the implementation of OH card interventions led to a noteworthy increase in positive coping scores among nurses (28). Additionally, their research highlighted that the OH card interventions positively impacted the post-traumatic growth scores of children with bone fractures, enhancing their coping strategies, reducing depressive symptoms, and ameliorating their overall psychological well-being (29). OH cards are perceived as a viable form of psychotherapy to assist patients in managing their emotional challenges (25). They enable clients to delve deeper into self-awareness by interpreting their experiences and articulating the origins of their most distressing post-traumatic stress disorder (PTSD) symptoms (29). OH cards offer a secure and confidential channel for emotional catharsis, aiding individuals in navigating their trauma and emotional struggles. Within group counseling settings, OH cards can foster interaction and sharing among participants, facilitating a deeper understanding of individual needs, emotions, and fostering the development of healthier interpersonal bonds. Consequently, OH cards serve as a potent counseling instrument that aids individuals in cultivating their cognitive abilities comprehensively.

Tu et al. and Nogalski et al. examined coping styles in patients with cancer and chronic and traumatic illnesses and found that positive coping styles can promote psychological recovery (33, 34). Positive coping styles reflect cognitive or behavioral strategies that individuals use when experiencing stress that can lead to positive outcomes, such as changing perceptions by using cognitive resources, actively problem solving, or seeking social support from family and friends (35). In contrast, negative coping styles reflect an individual’s tendency to use cognitive or behavioral strategies that can lead to negative outcomes when experiencing stress, such as cognitive avoidance, expressive inhibition and substance abuse (36). Nevertheless, the OH intervention failed to diminish negative coping responses associated with breast cancer, indicating that this therapeutic approach may not universally address negative cognitive patterns. This implies the necessity of considering alternative psychological strategies to effectively manage such responses.

In China, mental health issues have long been stigmatized (37),resulting in many patients’ reluctance to openly discuss their conditions or seek professional assistance. Cultural factors play an important role in influencing the rate and performance of stigma of mental illness (38), particularly within East Asian contexts where Confucian values emphasizing social harmony, the cultural primacy of “face” preservation, and stigmatizing folk beliefs regarding mental disorder etiology collectively contribute to heightened stigmatization patterns (39).While we hypothesize that disease-related stigma may exist among the patient cohort, and bias in the scale scores due to the concealment of true feelings by some patients, its potential impact on clinical outcomes remains inconclusive. Study strongly suggests cognitive behavioral therapy (CBT) as an effective non-pharmacological method for the treatment of psychiatric disorders of BC patients during cancer treatments and also for BC survivors (40). However, multiple systemic barriers significantly impede patient-initiated engagement with CBT delivery, including: Stigma-related barriers, cultural determinants as mentioned, and additional costs for medical care. In this study, the use of OH card is more intuitive and friendly to some people with lower level of cultural understanding, because of the role of pictures in improving health communication (41). The pictorial nature of OH cards facilitates cross-cultural resonance and subconscious projection, enabling psychotherapists to access patients’ psychological states across diverse cultural backgrounds. As cultural differences appear to be found primarily in the area of reduced positive affect in the Chinese patients, interventions that target positive affect may be particularly beneficial to improve QOL in Chinese women (42). OH card interventions provide a complement to mind–body therapies (e.g., meditation, yoga, tai chi) in breast cancer care, effectively addressing breast cancer-specific challenges, including post-surgical complications (e.g., lymphoedema), treatment-related fatigue, and body image concerns, while providing psychological benefits without requiring physical exertion.

Of course there are limitations to this study. ‌The primary methodological limitation stems from the non-randomized design introducing potential selection bias. Although no statistically significant differences in baseline characteristics were observed, the self-selection process for intervention allocation may affect the generalizability of the results. Subsequent research should prioritize randomized controlled trials with stratification by cancer stage (I-III) and metastasis status, or use matching methodologies (e.g., propensity score analysis) to enhance the reliability of causal inferences. Secondly, the small sample size may limit the ability to detect long-term effects (such as 3–6 months), especially when the effect size and power is small. Future studies should employ adequately powered sample sizes to detect OH card intervention effects on HADS outcomes and SCSQ, with *a priori*-defined primary/secondary endpoints to ensure methodological rigor. Finally, for some patients the OH card may be a completely new approach, some may be confused or not sure if they understand the card correctly, so it may take some time for them to adapt and become familiar with this counseling approach. While this study featured only one experiential activity, a more intricate and diverse array of interventions is essential to aid patients in ameliorating their negative emotional states. OH cards, as a novel group therapy instrument, lack a comprehensive and systematic theoretical framework akin to individual therapy. The credibility of these cards as therapeutic aids may be subject to scrutiny, given the limited research on their application in counseling contexts due to the scarcity of practitioners utilizing them. Consequently, it is imperative to conduct further research to impartially evaluate the efficacy of OH cards as counseling tools. Moreover, additional investigations are warranted to delineate the specific domains in which OH cards excel in delivering individual therapy to clients, thereby elucidating the true potential of OH cards.

## Conclusion

In conclusion, this study provides valuable insights regarding OH card interventions for breast cancer survivors. It has drawn attention to psychological status and coping styles when people experiencing BC, and suggests that female breast cancer survivors might benefit more from “OH cards” interventions.

## Data Availability

The original contributions presented in the study are included in the article/supplementary material, further inquiries can be directed to the corresponding author.
